# Acquired resistance to 5-fluorouracil *via* HSP90/Src-mediated increase in thymidylate synthase expression in colon cancer

**DOI:** 10.18632/oncotarget.5327

**Published:** 2015-09-23

**Authors:** Ji-Young Ahn, Ji-Sun Lee, Hye-Young Min, Ho-Young Lee

**Affiliations:** ^1^ College of Pharmacy and Research Institute of Pharmaceutical Sciences, Seoul National University, Seoul 151-742, Republic of Korea

**Keywords:** 5-fluorouracil, colon cancer, HSP90, Src

## Abstract

5-fluorouracil (5-FU), one of the first-line chemotherapeutic agents for the treatment of gastrointestinal malignancies, has shown limited efficacy. The expression of thymidylate synthase (TYMS) has been reported to be associated with the resistance to 5-FU. Here, we demonstrate that the enhanced HSP90 function and subsequent activation of Src induce expression of TYMS and acquired resistance to 5-FU in colon cancer. We show that the persistent 5-FU treatment granted 5-FU-sensitive HCT116 colon cancer cells morphologic, molecular, and behavioral characteristic of the epithelial-mesenchymal transition (EMT), contributing to emergence of acquired resistance to 5-FU. HCT116/R, a HCT116 colon cancer cell subline carrying acquired resistance to 5-FU, showed increased expression and activation of HSP90's client proteins and transcriptional up-regulation of TYMS. Forced overexpression of HSP90 or constitutive active Src in HCT116 cells increased TYMS expression. Conversely, pharmacological blockade of HSP90 or Src in HCT116/R cells effectively suppressed the changes involved in 5-FU resistance *in vitro* and xenograft tumor growth, hematogenous spread, and metastatic tumor development *in vivo*. This study suggests a novel function of HSP90-Src pathway in regulation of TYMS expression and acquisition of 5-FU resistance. Thus, therapeutics targeting this pathway may be an effective clinical strategy to overcome 5-FU resistance in colon cancer.

## INTRODUCTION

The antimetabolite drug 5-fluorouracil (5-FU) is widely used in the treatment of numerous cancers, including colorectal cancer [1]. 5-FU inhibits cancer cell growth by disrupting action of thymidylate synthase (TYMS), thereby causing DNA and RNA damage. Nonetheless, response rates to 5-FU are only 10 to 15% in colorectal cancer [2], and combination with other anti-cancer drugs, such as cetuximab, cisplatin and oxaliplatin, has improved response rates to 5-FU [1, 3–5]. Multiple mechanisms, including acquisition of epithelial mesenchymal transition (EMT) have been known to play a role in 5-FU resistance [1, 6, 7]. Histone deacetylase was also found to contribute to 5-FU resistance by increasing acetylation of HSP90, a key chaperone protein protecting several client oncoproteins, such as Src, ErbB2, Akt, and Raf, from proteosomal degradation [8].

Among resistance mechanisms reported so far, TYMS is known to play a central role in 5-FU resistance. Tumors with elevated TYMS have highly proliferative and metastatic characteristics [9, 10]. 5-FU sensitivity and patients’ survival have been inversely related to the level of TYMS protein and enzymatic activity in cancer cells, and 5-FU-resistant tumors commonly express high levels of TYMS protein [11]. Previous results have demonstrated that Src modulates TYMS expression [8, 12]. However, the detailed mechanisms underlying resistance to 5-FU and regulation of TYMS expression remain to be elucidated.

This study was performed to investigate the mechanism by which TYMS expression is regulated and colon cancer cells become resistant to 5-FU. We show that increased HSP90 function after chronic exposure to 5-FU leads to Src activation, resulting in induction of genotypic and phenotypic changes involved in the EMT, induction of TYMS expression, and 5-FU resistance in colon cancer cells. More importantly, treatment with clinically available inhibitors targeting Src or HSP90 was found to suppress primary tumor growth, circulation in the blood, and metastatic tumor development of 5-FU-resistant cancer cells *in vivo*. Our results suggest potential clinical benefit of targeting HSP90 or Src in colon cancer patients who acquired resistance to 5-FU-based therapeutic regimens.

## RESULTS

### *In vitro* selection of colon cancer cells with acquired 5-FU resistance

**Figure 1:**
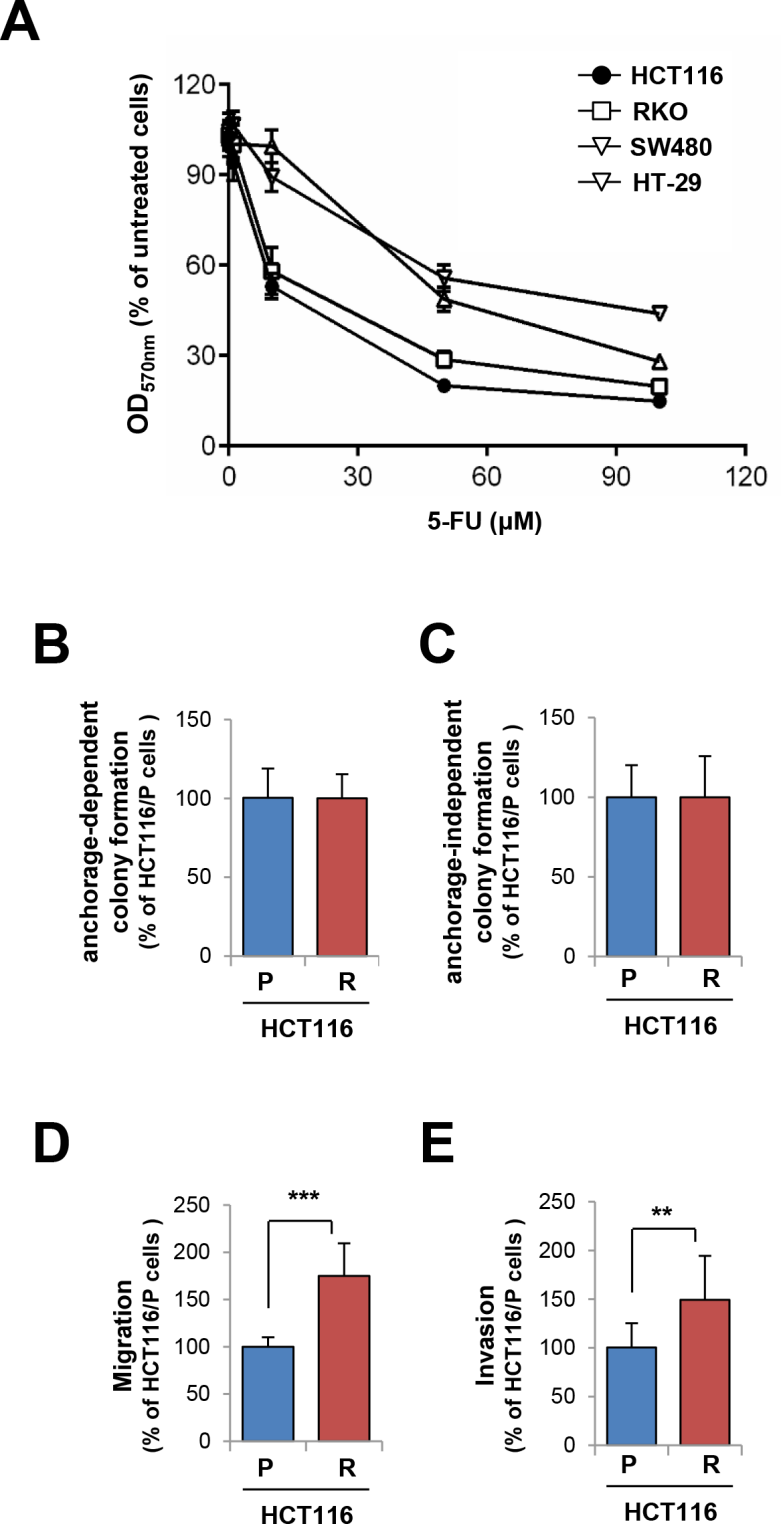
Generation and characterization of 5-FU resistant cells **A.** Colon cancer cells were treated with increasing concentrations of 5-FU for 3 days. Cell viability was determined by the MTT assay. **B–E.** Basal phenotype alterations including anchorage-dependent (B) and -independent colony formation (C), migration (D), and invasion (E) of 5-FU-resistant HCT116 cells (HCT116/R) compared with parental cells (HCT116/P) were determined. **P < 0.01; ***P < 0.001.

We selected 5-FU-sensitive HCT116 human colon cancer cells based on the results from a (3-[4,5-dimethylthiazol-2-yl]-2,5 diphenyl tetrazolium bromide (MTT) assay (Figure 1A). We established a subline of HCT116 cells carrying acquired resistance to 5-FU by treating the cell line with increasing concentrations of 5-FU over a period of more than 6 months. Compared to the parental cells (HCT116/P), resistant cells (HCT116/R) exhibited minimal change in anchorage-dependent (Figure 1B) and -independent (Figure 1C) colony forming abilities but significantly greater migration (Figure 1D) and invasion (Figure 1E).

**Figure 2:**
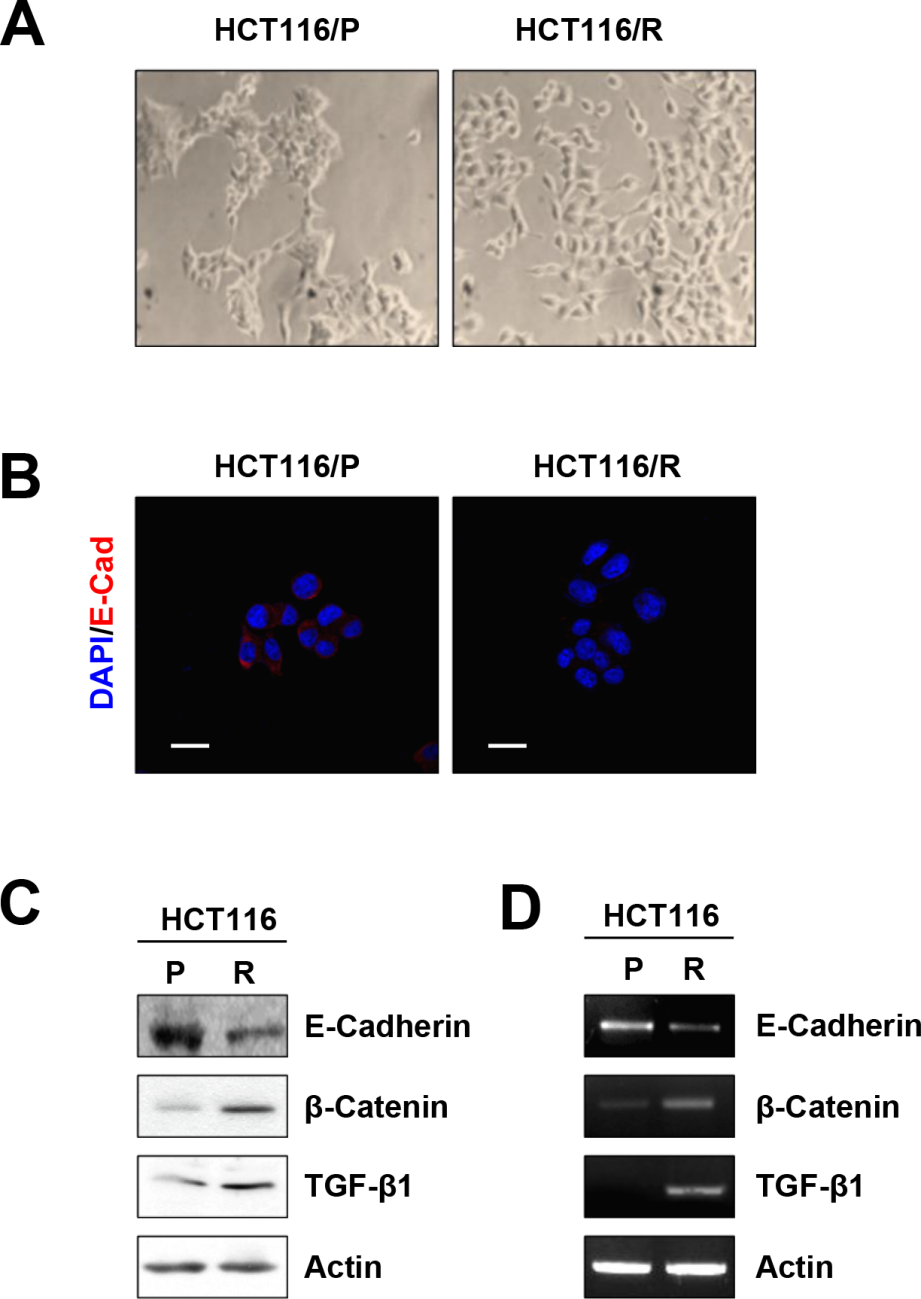
Acquisition of EMT phenotype in 5-FU resistant cells **A.** Morphological changes were examined using a phase-contrast microscope. **B.** immunofluorescence staining of each cells using anti-E-cadherin (Scale bar: 20 μm, 400× magnifications). **C.** E-cadherin, β-catenin, and TGF-β1 expression in each cells was determined by Western blot. **D.** The expression of E-cadherin, β-catenin, and TGF-β1 mRNA in each cells was analyzed by RT-PCR.

Notably, HCT116/P cells exhibited a cobblestone-like morphology with tight cell–cell junctions, whereas HCT116/R cells displayed spindle-like and elongated fibroblastic cell morphology with loss of intercellular adhesion and increased pseudopodia (Figure 2A). Immunofluorescence staining (Figure 2B), Western blotting (Figure 2C), and RT-PCR analysis (Figure 2D) revealed that HCT116/R cells exhibited reduced E-cadherin and increased β-catenin and TGF-β1 expression. Collectively these results suggest that EMT is related to acquisition of resistance to 5-FU.

**Figure 3:**
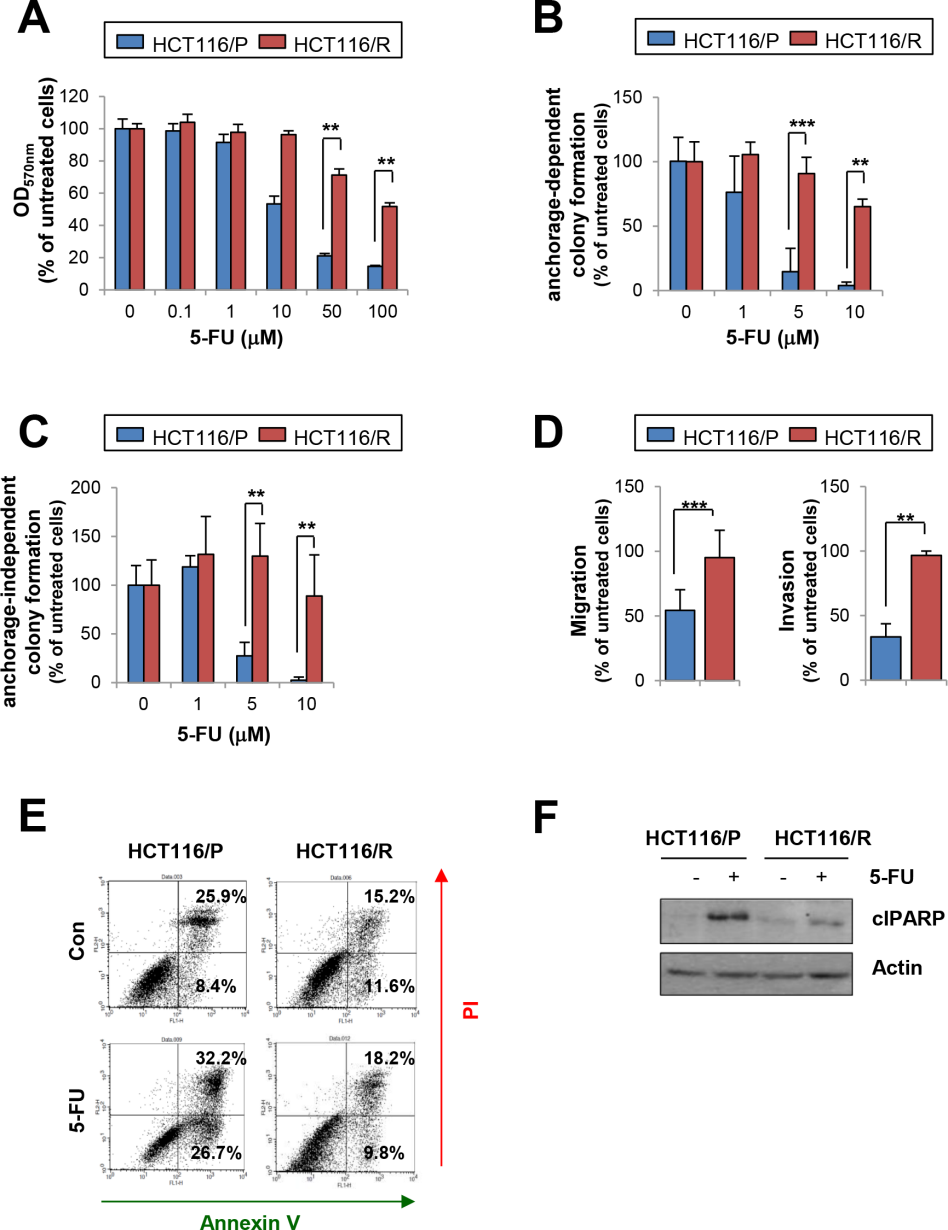
Effects of 5-FU on HCT116/P and HCT116/R cells **A.** Cell viability was determined by the MTT assay after 3 days of 5-FU treatment. **B, C.** The effect of 5-FU on anchorage-dependent (B) and -independent (C) colony formation in HCT116/R cells compared with HCT116/P cells was determined. **D.** Migration and invasion of HCT116/P and HCT116/R cells with 5-FU treatment was compared with untreated cells. **E, F.** Resistance to 5-FU-induced cell death in HCT116/R cells. HCT116/P and HCT116/R cells were treated with 5-FU (50 μM) for 2 days. (E) Increases in apoptotic cell populations were determined by flow cytometry after staining unfixed cells with annexin V and PI. (F) PARP cleavage modulation upon 5-FU treatment was determined by Western blot. **P < 0.01; ***P < 0.001.

We next tested the effects of 5-FU on HCT116/P and HCT116/R cells. Following 5-FU treatment, HCT116/R cells exhibited significantly greater viability (Figure 3A), anchorage-dependent (Figure 3B), -independent colony formation (Figure 3C), migration and invasion (Figure 3D) compared with the parental cells. 5-FU-induced apoptosis was also blocked in HCT116/R cells as determined by annexin V-propidium iodide (PI) double staining (Figure 3E) and cleavage of poly (ADP-ribose) polymerase (PARP) (Figure 3F).

### Enhanced HSP90 function and subsequent Src activation increases TYMS expression in 5-FU-resistant cells

**Figure 4:**
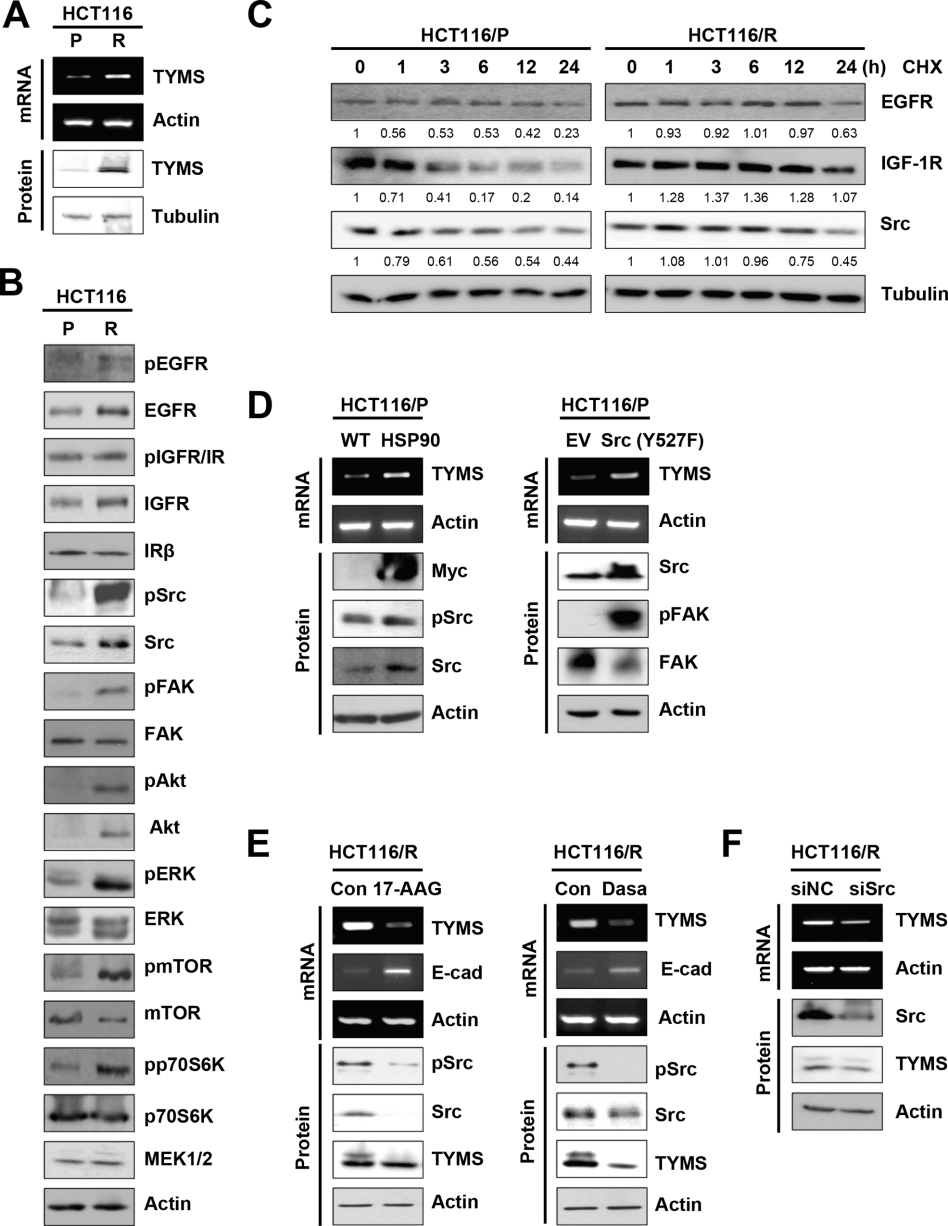
Activation of the HSP90-mediated Src signaling, contributing to increase in TYMS expression **A.** Western blot and RT-PCR analysis for TYMS level in HCT116/P and HCT116/R cells. **B.** Changes in various signaling pathways in HCT116/R cells compared with HCT116/P cells were analyzed by Western blot. **C.** Cells were exposed to cycloheximide (CHX, 100 μg/ml) for indicated times, and protein lysates were harvested. EGFR, IGF-1R, and Src protein expression was determined by Western blot. Numbers below the blot indicate densitometric quantification relative to 0 h treatment. **D.** HCT116/P cells were transfected with HSP90 (left) or constitutively-active Src {Src(Y527F); right} and expression of TYMS were detected by RT-PCR. **E.** HCT116/R cells were treated with 17-AAG (100 nM; left) or dasatinib (50 nM; right) for 48 h and expression of TYMS, E-cadherin, and Src phosphorylation were detected by RT-PCR or Western blot. (E, right) Src expression was reduced using siRNA in HCT116/R cells, and expression of TYMS was determined by RT-PCR or Western blot.

5-FU resistance has been attributed to overexpression of TYMS [1, 12, 13]. Consistently, we found greater levels of TYMS mRNA and protein expressions in HCT116/R cells than in HCT116/P cells (Figure 4A). We then assessed the mechanisms underlying increased levels of TYMS expression along with acquisition of EMT phenotypes and 5-FU resistance in HCT116/R cells. Because the EMT process can be regulated by a diverse array of cytokines and growth factors [14], we analyzed the activation status of several kinases and their effectors involved in cancer cell proliferation and survival, including EGFR, IGF-1R, Src, FAK, Akt, Erk1/2, mTOR, MEK1/2, and p70S6K. Compared to HCT116/P cells, HCT116/R cells were found to have increased expression and phosphorylation of EGFR, IGF-1R, Src, and Akt (Figure 4B). Increased phosphotylations of Erk, mTOR, and p70S6K were also detected in HCT116/R cells compared to their parental cells. Notably, mRNA levels of EGFR, IGF-1R, Src, Akt, and mTOR remained unchanged in HCT116/R cells (Supplementary Figure S1).

Based on the given role of the molecular chaperone HSP90 in the stability of EGFR, IGF-1R, Src, and Akt proteins, we reasoned that HSP90 function might be involved in increased levels of these proteins in HCT116/R cells. Indeed, HCT116/R cells revealed an increased half-life of HSP90 client proteins, including EGFR, IGF-1R, and Src proteins (Figure 4C). Because Src activity was found to regulate TYMS transcription [12], we further hypothesized that increased HSP90 function and subsequent activation of Src could have contributed to TYMS expression in HCT116/R cells. Indeed, forced HSP90 overexpression led to increased levels of TYMS, Src and pSrc expressions in HCT cells (Figure 4D, left). Conversely, treatment of HCT116/R cells with the HSP90 inhibitor 17-AAG induced a transcriptional decrease in TYMS expression (Figure 4E, left). Moreover, activation of Src by transfection with constitutively active Src (Y527F) resulted in FAK activation along with a transcriptional increase in TYMS in HCT116/P cells (Figure 4D, right). Conversely, treatment with dasatinib, a Src inhibitor decreased TYMS expression in HCT116/R cells (Figure 4E, right). Considering that dasatinib inhibits a wide variety of tyrosine kinases [15], we induced knockdown of Src by using siRNA to show the direct impact of this kinase in regulating TYMS expression. Upon knockdown of Src expression, TYMS expression was also decreased in HCT116/R cells (Figure 4F), supporting that Src regulates the TYMS expression. Additionally, pharmacological inhibitors of Akt (LY294002) or EGFR (erlotinib) also decreased the expression of TYMS in HCT116/R cells (Supplementary Figure S2). Inhibition of HSP90 or Src increased transcription of E-cadherin (Figure 4E), indicating that induction of EMT in resistant cells was originated from increased HSP90 and Src activities. These findings suggest that activation of the HSP90-mediated Src signaling pathway was stimulated in HCT116/R cells, resulting in increase of TYMS expression.

### Targeting HSP90 or Src suppresses primary tumor growth and metastatic lung tumor development of 5-FU resistant colon cancer cells *in vivo*

**Figure 5:**
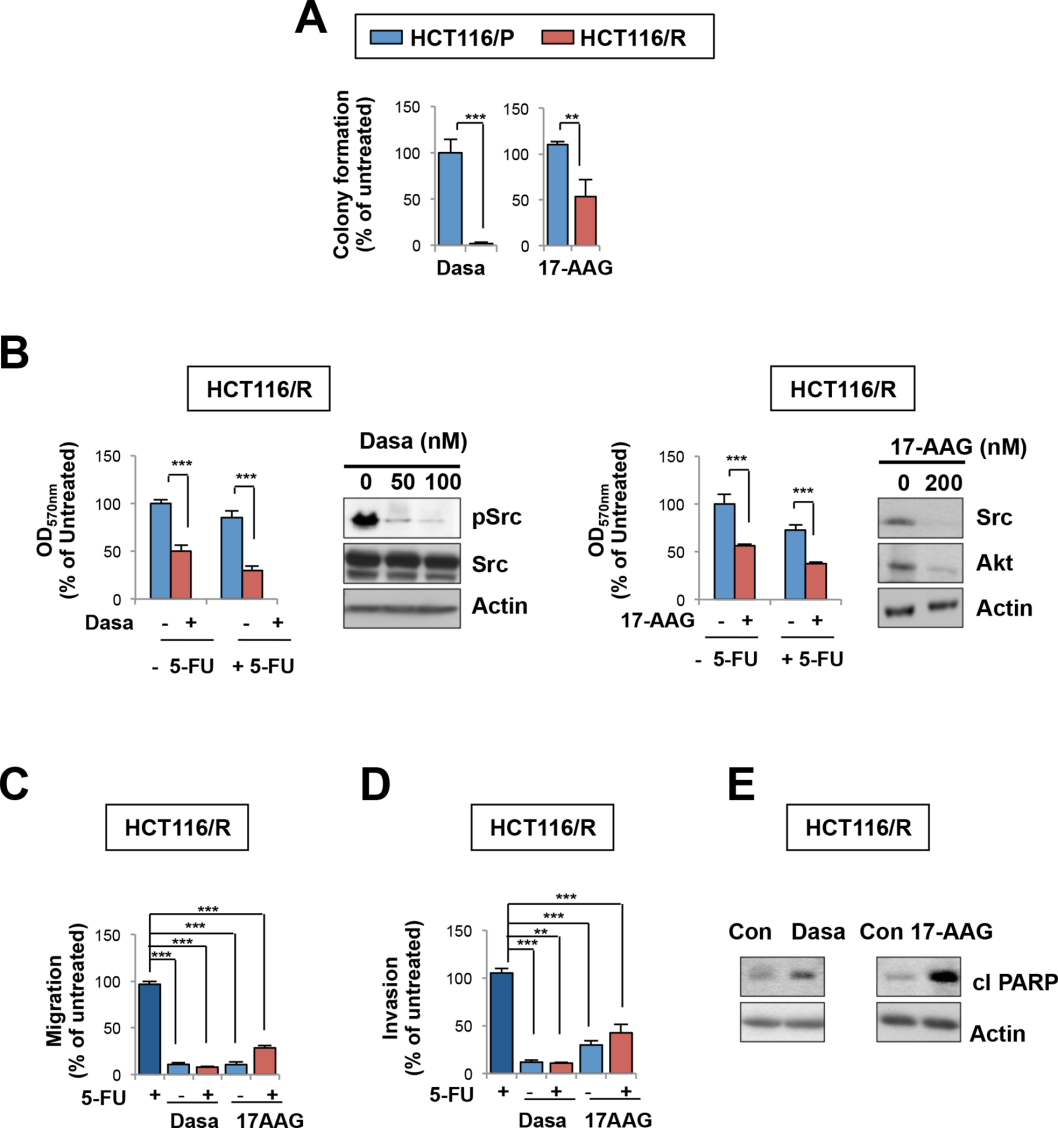
HSP90 or Src blockade suppresses growth, migration, and invasion of 5-FU resistant cells **A.** Anchorage-independent colony formation of HCT116/P and HCT116/R cells under dasatinib or 17-AAG treatment. **B.** HCT116/R cells were treated with 5-FU alone or in combination with dasatinib (100 nM) or 17-AAG (200 nM), and cell viability was determined by the MTT assay. Relative cell viability was presented as percentage of untreated cells. The effect of each inhibitor on the regulation of its target signaling was determined by Western blot. **C, D.** The effects of 5-FU combined with dasatinib or 17-AAG on HCT116/R cell migration (C) and invasion (D) **P < 0.01; ***P < 0.001. **E.** Cleaved PARP under dasatinib or 17-AAG in HCT116/R cells was detected by Western blot.

We explored whether blockade of HSP90-Src pathway is an effective anticancer strategy in 5-FU-resistant tumors. Suppression of Src or HSP90 in HCT116/R cells by each inhibitor significantly suppressed colony formation (Figure 5A), viability (Figure 5B), migration (Figure 5C), and invasion (Figure 5D). Apoptosis was also induced by the treatment as shown by induced PARP cleavage (Figure 5E). Notably, co-treatment with 5-FU appeared to have minimal benefit in enhancing the antitumor effects of each inhibitor (Figures 5B, 5C and 5D), suggesting that HSP90 or Src blockade would effectively suppress growth, migration, and invasion of 5-FU resistant colon cancer cells. Akt inhibition by the LY294002 treatment also suppressed colony forming ability, viability, and migration with minimal benefit in enhancing antitumor activities of 5-FU in HCT116/R cells. (Supplementary Figure S3). These results imply that HCT116/R cells are dependent on HSP90 function for various tumor activities.

To investigate the impact of HSP90 and Src in innate resistance in colon cancer cells, we compared basal levels of target proteins of HSP90 in HCT116, RKO, HT-29, and SW480 cells. Among those cell lines, SW480 cells, the most resistant cell line according to Figure 1A, showed the strongest expression of Src and Akt proteins (Supplementary Figure S4A). When Src was targeted by dasatinib treatment, viability of SW480 cells remained unaffected. In contrast, combined treatment with 5-FU and dasatinib significantly enhanced the inhibitory effects of 5-FU on the viability of SW480 cells (Supplementary Figure S4B). This data suggest that HSP90/Src pathway is implicated in the innate resistance to 5-FU in colon cancer cells.

**Figure 6:**
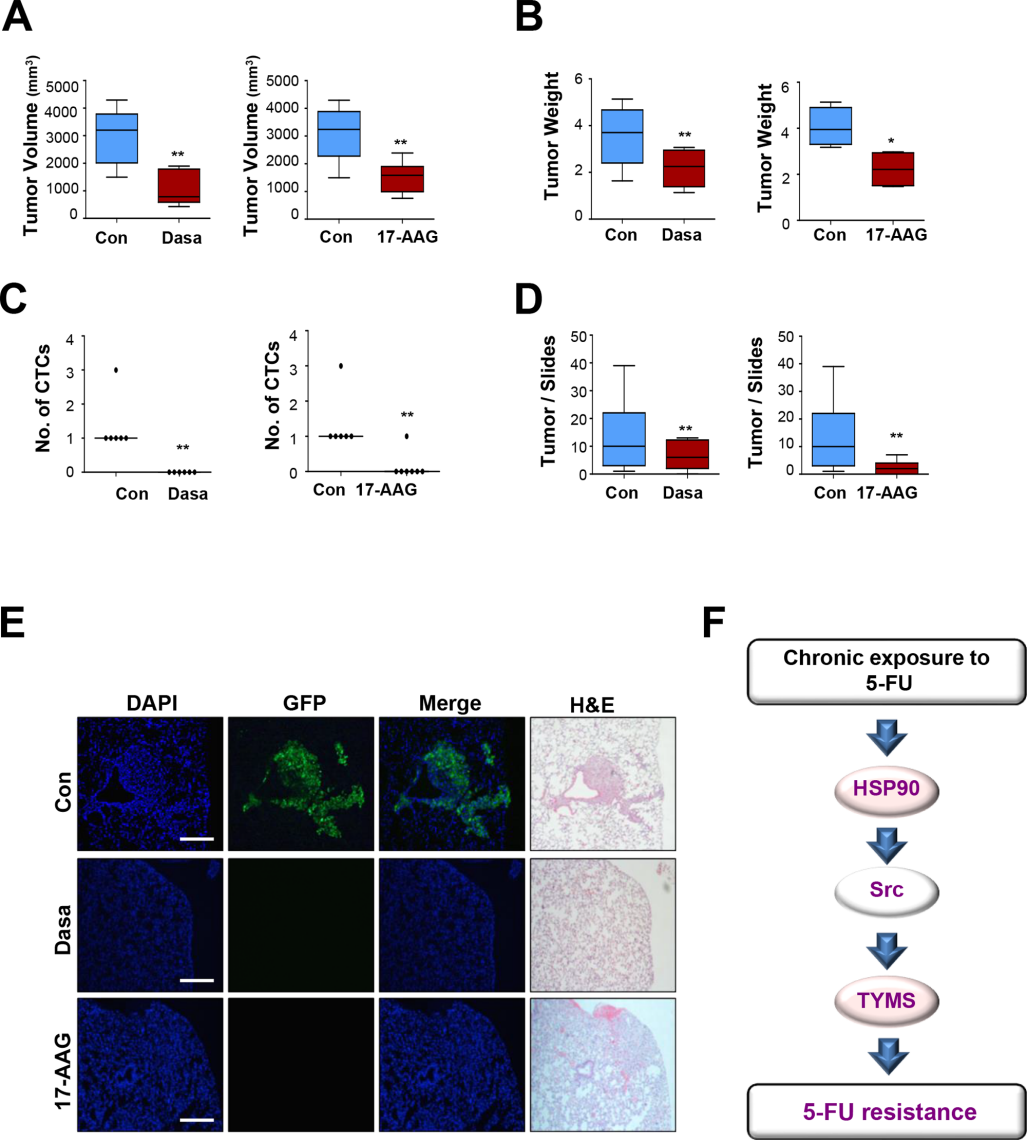
HSP90 or Src inhibition decreases primary tumor growth, the number of circulating tumor cells, and lung metastasis **A–E.** Effects of HSP90 or Src inhibition in renal capsule implantation model of HCT116/R cells. The volume (A) and weight (B) of primary tumors derived from cells implanted under the renal capsule were examined after 3 weeks of drug treatment; dasatinib (70 mg/kg, oral gavage) or 17-AAG (70 mg/kg, intraperitoneal injection) were administered to mice 5 days per week. (C) The dissemination of implanted cells in the circulation in each treatment group was determined by observation of smeared blood under a fluorescence microscope and the enumeration of circulating tumor cells is shown. CTC = Circulating Tumor Cell. (D) Quantification of metastatic lung tumor nodule formation in each treatment group compared with the vehicle-treated control group. **P < 0.01; ***P < 0.001. (E) Lung metastases were decreased upon HSP90 or Src inhibition as determined by observation under a fluorescence microscope. Lung tissue specimens stained with hematoxylin and eosin were observed under an inverted microscope to confirm metastatic tumor formation (Scale bar: 50 μm, 100 × magnifications). *N* = 5 to 7 per group. **F.** Schematic illustration of the relationships among HSP90, Src, and TYMS in 5-FU resistance. Upon chronic exposure to 5-FU, enhanced HSP90 function results in Src activation followed increase in TYMS expression, and this leads 5-FU resistance by activating cancer cell proliferation, survival, migration, and invasion.

To explore the effects of HSP90 and Src antagonism on tumor growth and metastatic tumor development *in vivo*, we performed renal capsule implantation of GFP-labeled HCT116/R cells. We have previously shown that HCT116/R cells implanted under the renal capsule induced an obvious increase in the number of circulating tumor cells within 5 weeks, leading to metastatic tumor growth [16]. We observed a statistically significant difference in volume (Figure 6A) and weight (Figure 6B) of the primary tumors between the vehicle- and each inhibitor-treated groups. Tumor nodules were detected in the lung sections obtained from mice with HCT116/R cell implantation. We also observed that dissemination of circulating HCT116/R cells (Figure 6C) and metastatic lung tumor nodules were significantly decreased in mice treated with each inhibitor (Figures 6D and 6E). These *in vivo* results suggest that targeting the HSP90-Src-mediated pathway may effectively suppress tumor growth and metastasis of 5-FU resistant colon cancer, providing effective second-line treatment strategies for colon cancer.

## DISCUSSION

Because 5-FU still remains the agent of choice in the treatment of GI tract cancers, identifying key pathways that cause 5-FU resistance and developing effective therapeutic strategies for the patients who acquired resistance against 5-FU-based therapies are of vital importance. In the current study, we show potential mechanisms of 5-FU-resistance using a 5-FU sensitive parental cell line (HCT116/P) and its subline (HCT116/R) with acquired resistance to 5-FU. The increased migratory and invasive potential and morphological alterations observed in HCT116/R cells are typical characteristics of the EMT, which contributes to the emergence of cancer stem cells and drug resistance [17]. Therefore, it can be suggested that induction of EMT results in 5-FU resistant phenotype in HCT116 cells. We also show that HSP90-dependent Src activation plays a key role in the changes observed in HCT116/R cells. We finally show that HSP90 and Src antagonism are highly effective in suppressing TYMS expression and primary tumor growth, dissemination in circulation, and metastatic tumor formation of the HCT116/R cells. Based on these results, we propose that HSP90-dependent Src activation after chronic exposure to 5-FU causes increase in TYMS expression in both mRNA and protein levels, leading to 5-FU resistance. A model of how chronic exposure to 5-FU leads to increase in TYMS expression and drug resistance is described in Figure 6F. These findings indentify a previously unrecognized role of HSP90-mediated Src activation in TYMS expression and acquired resistance to 5-FU in colon cancer.

HCT116/R cells revealed several molecular and behavioral changes associated with EMT, including (a) formation of spindle-shaped cells and pseudopodia; (b) transcriptional regulation of representative markers; (c) enhanced invasive and migratory activities; and (d) increased expression, stability and/or activation of several HSP90 client proteins (Src, EGFR, IGF-1R, and Akt) and their downstream signaling mediators (FAK, Erk1/2 and mTOR) along with increased level of TYMS expression. HCT116/R cells appeared to have markedly increased levels of Src activity through enhanced function of HSP90. Those changes suggest that chronic 5-FU exposure selected resistant cells which acquire survival mechanism through activation of oncogenic signaling molecule, including Src. This is in line with previous discussions that resistance of therapy occurs by killing sensitive cells and sparing mutant cells that are resistant [18–20]. Studies have identified a role of extracellular HSP90 and Src in driving EMT, a key step implicated in metastasis and resistance to chemotherapy, radiation therapy, and various molecularly targeted therapies in diverse preclinical models [21–25]. Previous studies also suggest that Src may induce 5-FU resistance by interfering with several signaling molecules, including PI3K, Akt, and NF-κB [26, 27]. Moreover, HSP90 inhibitors exhibited a potent anti-proliferative effect in various 5-FU-resistant prostate cancer cells [28]. Our findings are relevant to and extend these earlier reports that identify roles for HSP90 and Src in driving EMT and 5-FU resistance [8, 12, 21, 22]. Therefore, it was likely that 5-FU resistance emerged at least in part by EMT through enhanced HSP90 function and subsequent increases in multiple oncogenic HSP90 client proteins. Hence, targeting HSP90 or Src could be clinically available strategies to overcome 5-FU chemoresistance.

Notably, increased TYMS expression was detected in HCT116/R cells compared to HCT116/P cells. TYMS, which is involved in the metabolic process of 5-FU has been shown to mediate 5-FU resistance and predict prognosis of patients with colon cancer [29]. It was previously reported that inactivation of Src kinase could revert chemoresistance to TYMS-targeting anticancer drugs [12]. Histone deacetylase inhibition was also found to reverse 5-FU resistance by decreasing *TYMS* mRNA expression and by inducing proteasomal degradation of the TYMS protein *via* HSP90 acetylation [8]. Moreover, HSP90-targeting small molecule inhibitor, ganetespib, was shown to downregulate TYMS, thus sensitizing colorectal cancer cell line to 5-FU [30]. These previous reports and our *in vitro* findings from the current study, including 1) increase in HSP90 level or constitutive activation of Src increase TYMS expression and 2) pharmacologic suppression of HSP90 or Src decreases TYMS mRNA and protein expression suggest that up-regulation of TYMS expression in HCT116/R cells were through enhanced HSP90 and Src activities and might be a fundamental mechanism by which colon cancer cells acquired EMT phenotypes and 5-FU resistance. In support of the notion, pharmacologic suppression of Src or HSP90 abolished TYMS expression, colony forming abilities, potentials of migration and invasion and induces apoptosis in HCT116/R cells. This conclusion was further supported by our *in vivo* findings using the xenograft and renal capsule tumors established in nude mice that HSP90 or Src antagonism alone was sufficient to suppress HCT116/R cells’ primary tumor growth, hematogenous spread, and distal metastatic potential.

Interestingly, co-treatment with HSP90 or Src inhibitor showed no benefit to enhance 5-FU sensitivity. This can be explained by the fact that decreased TYMS upon 17-AAG or dasatinib is a target of 5-FU. Although 5-FU does not decrease the protein level, it inhibits the function of TYMS, and this also obtained by reducing the protein expression using 17-AAG and dasatinib. As the final effects of these inhibitors are same and activated HSP90 and Src in resistant cell line, single treatment of 17-AAG or dasatinib is enough to overcome the resistance to 5-FU.

In conclusion, this study identifies transcriptional overexpression of TYMS through the activation of an HSP90–Src signaling module as a novel mechanism for acquired resistance to 5-FU. Therefore, targeted inactivation of HSP90 and Src with clinically available inhibitors might be translated into the clinics for the patients with 5-FU resistance. In this context, our findings have therapeutic impact considering ongoing clinical development of HSP90 and Src inhibitors. Further clinical investigations are warranted to investigate whether such therapeutic regimens are effective in patients with 5-FU–resistant colorectal cancers. In addition, the exploration of detailed mechanisms underlying Src-mediated TYMS regulation are warranted.

## MATERIALS AND METHODS

### Reagents

Dasatinib and Erlotinib were purchased from LC Laboratories (Woburn, MA, USA), 17-AAG and LY294002 were purchased from EMD Chemicals (Gibbstown, NJ, USA), respectively. Unless otherwise indicated, chemicals were purchased from Sigma-Aldrich (St. Louis, MO, USA). The constitutively active Src mutant (Src (Y527F)) expression vector was kindly provided by Dr. Faye M. Johnson (MD Anderson Cancer Center, Houston, TX, USA). The HSP90 expression vector was kindly provided by Dr. William C. Sessa (Yale University School of Medicine, CT, USA). siRNA targeting Src was purchased from Dharmacon.

### Cell culture

The human colon cancer cell lines HCT116, RKO, SW480, and HT-29 were kindly provided by Dr. Sang Kook Lee (Seoul National University, Seoul, Republic of Korea). 5-FU resistant HCT116 (HCT116/R) cells were generated by continuous exposure to increasing concentrations of 5-FU for more than 6 months. All cell lines were cultured in RPMI 1640 media (Welgene, Daegu, Republic of Korea) supplemented with 10% FBS (Welgene) and 1% antibiotics (Welgene). Cells were incubated at 37°C in a 5% CO_2_ humidified atmosphere.

### MTT assay

MTT assay was performed as described previously [31]. Six replicate wells were used for each analysis, and three independent experiments were done.

### Anchorage-dependent and -independent colony formation analysis

For the anchorage-dependent colony formation analysis, 300 cells seeded on 6-well plates were treated with various inhibitors for 7–9 days. The colonies were fixed with methanol, stained with hematoxylin, and counted. Anchorage-independent colony formation analyses were done as described previously [32]. Independent experiments were repeated three times.

### Annexin V/PI double staining

Adherent and floating cells were collected, washed with PBS, diluted with 1X binding buffer (1 × 10^5^ cells/0.1 ml) and then stained using an annexin V/PI double staining kit (BD Biosciences) according to the manufacturer's instruction. The stained cells were analyzed using a FACSCalibur^®^ flow cytometer (BD Biosciences).

### Migration and invasion assay

Migration and invasion assays were performed as described previously [33]. The numbers of cells in four independent fields of triplicate were counted. Independent experiments were repeated three times.

### Western blotting

Total protein isolation and Western blotting analyses were done as described previously [32] with specific antibodies against the following antigens: pIGF-1R/IR (Y1131), IR, pEGFR (Y845), Src, pSrc (Y416), pp70S6K, p70S6K, pmTOR, mTOR, pAkt, Akt, pERK1/2 (p42/44), MEK1/2, ERK, tubulin (Cell Signaling Technology, Danvers, MA, USA); PARP, pFAK (Y397), and FAK (BD Biosciences, San Jose, CA, USA); Actin, IGF-1R (C-20), EGFR (1005), and Myc (9E10) (Santa Cruz Biotechnology, Santa Cruz, CA, USA); TYMS (Invitrogen, Carlsbad, CA, USA).

### Reverse transcription polymerase chain reaction (RT-PCR)

Total RNA extraction and reverse transcription was done as described previously [33]. PCR was performed using the primers described in Supplementary Table S1.

### Immunofluorescent staining

Immunofluorescent staining was performed as described previously [31].

### Animal model

All animal procedures were performed in accordance with a protocol approved by the Seoul National University Institutional Animal Care and Use Committee. Mice were fed standard mouse chow and water *ad libitum* and housed in temperature- and humidity-controlled facilities with a 12 h light/12 h dark cycle. Renal capsule implantation was performed as described previously [16]. 3 weeks after the implantation, mice were treated with dasatinib (70 mg/kg, 5 days a week by oral gavage) or 17-AAG (70 mg/kg, 5 days a week by intraperitoneal injection) for 2 weeks and sacrificed.

### Statistical analysis

Statistical comparisons between two groups were performed with unpaired Student's *t*-test using Microsoft Excel Software (Microsoft Corp., Redmond, MA, USA) or GraphPad Prism Software (GraphPad Software Inc., La Jolla, CA, USA), and two-sided *P*-values < 0.05 were considered statistically significant. Data are presented as means ± SD of a representative of at least three independently performed experiments.

## SUPPLEMENTAL FIGURES AND TABLE


