# Supplementary material for: Acquired resistance to 5-fluorouracil via HSP90/Src-mediated increase in thymidylate synthase expression in colon cancer
Source: Oncotarget. 2015 Sep 23;6(32):32622–33. doi: 10.18632/oncotarget.5327 (PMC4741717; doi:10.18632/oncotarget.5327)
Supplement: Supplementary file 1 [file oncotarget-06-32622-s001.pdf]

SUPPLEMENTAL FIGURES AND TABLE

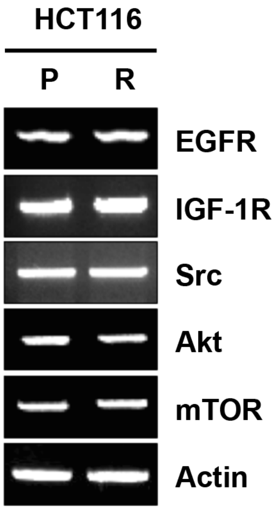

Supplementary Figure S1: Basal EGFR, IGF-1R, Src, Akt, and mTOR mRNA expression in HCT116/P and HCT116/R cells. Gene expression was analyzed by RT-PCR.

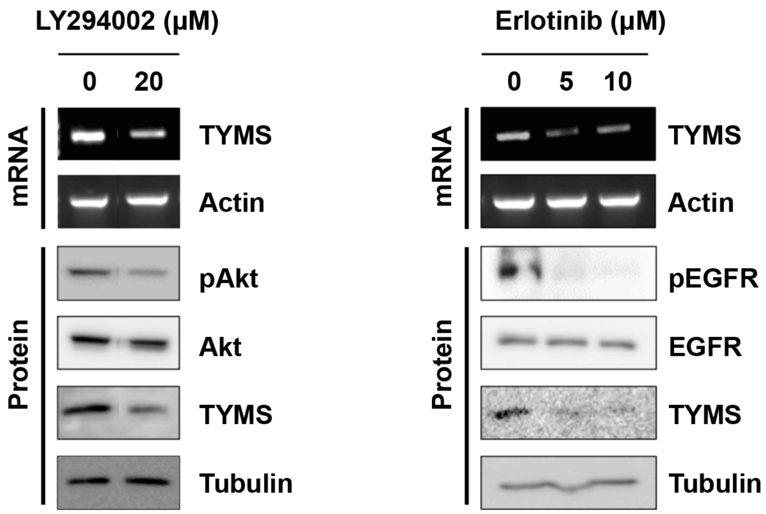

Supplementary Figure S2: Effects of inhibition of Akt or EGFR on expression of TYMS in HCT116/R cells. HCT116/R cells were treated with each inhibitors for 48 h and expression of TYMS were analyzed by RT-PCR and Western blot.

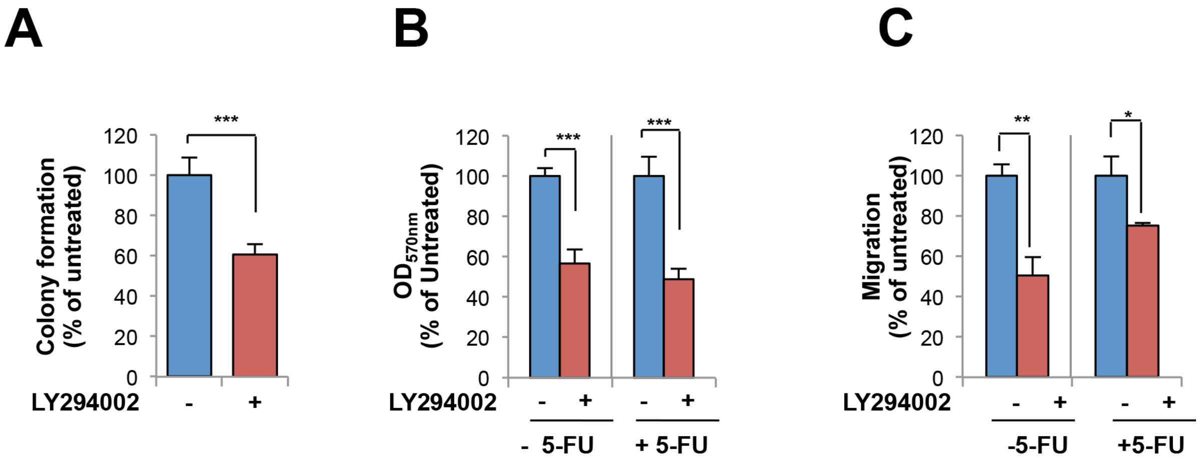

Supplementary Figure S3: Effects of Akt inhibition on viability, proliferation, and migration of HCT116/R cells. A. Anchorage-independent colony formation of HCT116/P and HCT116/R cells under LY294002 treatment. **B.** HCT116/R cells were treated with 5-FU alone or in combination with LY294002 (20 μM) and cell viability was determined by the MTT assay. Relative cell viability was presented as percentage of LY294002-untreated cells. **C.** The effects of 5-FU combined with LY294002 on HCT116/R cell migration. Relative cell migration was presented as percentage of LY294002-untreated cells. *$P < 0.05$; **$P < 0.01$; ***$P < 0.001$.

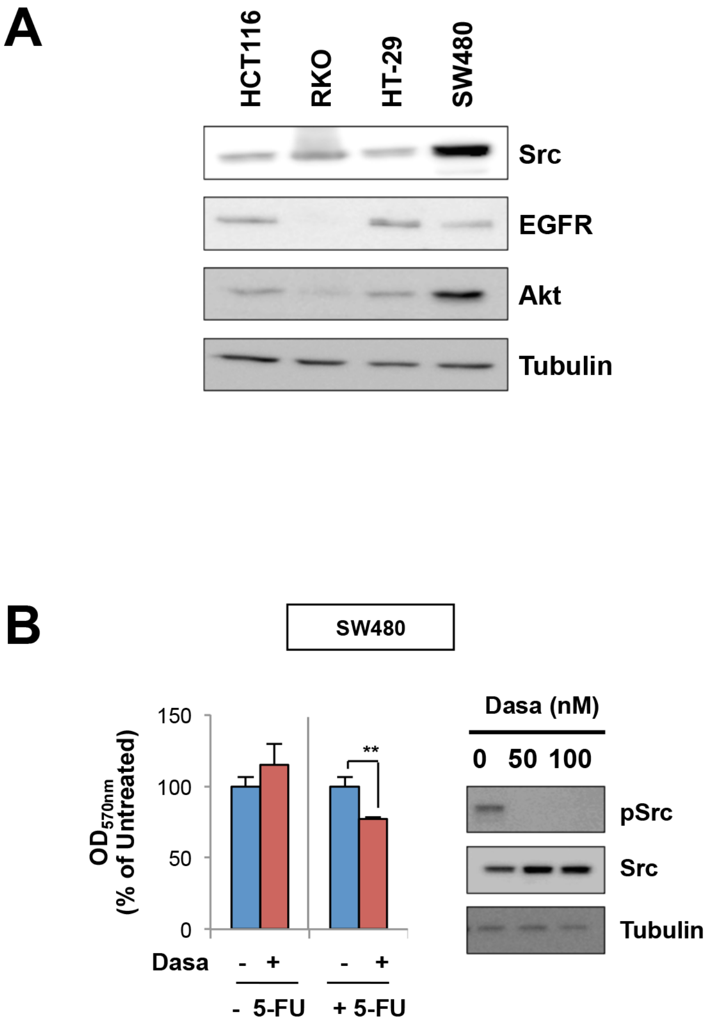

Supplementary Figure S4: Involvement of HSP90-Src pathway in innate resistance to 5-FU in SW480 cells. A. Basal levels of each proteins in indicated colon cancer cell lines were determined by Western blot. **B.** SW480 cells were treated with 5-FU alone or in combination with Dasatinib (50 nM) and cell viability was determined by the MTT assay (left). Relative cell viability was presented as percentage of dasatinib-untreated cells. The effect of dasatinib on the regulation of Src phosphorylation was determined by Western blot (right).

Supplementary Table S1: PCR primer sequences used in this study

Gene	Direction	Sequence
TGF-β1	Forward	GGA CTA TCC ACC TGC AAG AC
	Reverse	CGG AGC TCT GAT GTG TTG AA
E-cadherin	Forward	GGC CAG CCA TGG GCC CTT GG
	Reverse	CAC CTT CAG CCA ACC TGT TT
β -catenin	Forward	GCT GAT TTG ATG GAG TTG GAC ATG G
	Reverse	GCC AAA CGC TGG ACA TTA GTG G
Actin	Forward	ACT ACC TCA TGA AGA TC
	Reverse	GAT CCA CAT CTG CTG GAA
TYMS	Forward	GGGCAGATCCAACACATCC
	Reverse	GGTCAACTCCCTGTCCTGAA
EGFR	Forward	TGG AGC TAC GGG GTG ACC GT
	Reverse	GGT TCA GAG GCT GAT TGT GAT
IGFR	Forward	TGG GCC AAG AGT GAG ATC
	Reverse	GTA TTC AGC CTC CTC CTT C
Src	Forward	ATC ACC GCA AGA GCT ACC AT
	Reverse	TGA CGG TGT CCG AGG AGT TG
Akt	Forward	ATG AGC GAC GTG GCT ATT GTG AAG
	Reverse	GAG GCC GTC AGC CAC AGT CTG GAT G
mTOR	Forward	GAA CGC TTG GCA GCT TTG AA
	Reverse	CAT GTA GGG GCG GAT GAG TC
